# Mycophenolic Acid for Topical Immunosuppression in Vascularized Composite Allotransplantation: Optimizing Formulation and Preliminary Evaluation of Bioavailability and Pharmacokinetics

**DOI:** 10.3389/fsurg.2018.00020

**Published:** 2018-05-09

**Authors:** Firuz G. Feturi, Matthias Weinstock, Wenchen Zhao, Wei Zhang, Jonas T. Schnider, Vasil E. Erbas, Sinan Oksuz, Jan A. Plock, Lisa Rohan, Alexander M. Spiess, Lydia M. Ferreira, Mario G. Solari, Raman Venkataramanan, Vijay S. Gorantla

**Affiliations:** ^1^Department of Pharmaceutical Sciences, School of Pharmacy, University of Pittsburgh, Pittsburgh, PA, United States; ^2^Disciplina de Cirurgia Plástica, Escola Paulista de Medicina, Universidade Federal de São Paulo, São Paulo, Brazil; ^3^Magee-Womens Research Institute, Pittsburgh, PA, United States; ^4^Division of Plastic and Hand Surgery, University Hospital Zurich, Zurich, Switzerland; ^5^Department of Plastic Surgery, Medicalpark Gaziantep Hastanesi, Gaziantep, Turkey; ^6^Department of Plastic Reconstructive and Aesthetic Surgery, Gulhane Medical School, Ankara, Turkey; ^7^Department of Plastic and Reconstructive Surgery, School of Medicine, University of Pittsburgh, Pittsburgh, PA, United States; ^8^McGowan Institute for Regenerative Medicine, Pittsburgh, PA, United States; ^9^Wake Forest Institute for Regenerative Medicine, Wake Forest Baptist Medical Center, Winston-Salem, NC, United States

**Keywords:** mycophenolic acid, topical, formulation, permeability, immunosuppression, VCA

## Abstract

Mycophenolic acid (MPA), is the active form of the ester prodrug mycophenolate mofetil (MMF). MMF is an FDA approved immunosuppressive drug that has been successfully used in systemic therapy in combination with other agents for the prevention of acute rejection (AR) following solid organ transplantation (SOT) as well as in vascularized composite allotransplantation (VCA). Systemic use of MMF is associated with gastrointestinal adverse effects. Topical delivery of the prodrug could thus provide graft-targeted immunosuppression while minimizing systemic drug exposure. Our goal was to develop a topical formulation of MPA with optimal *in vitro*/in vivo characteristics such as release, permeation, and tissue bioavailability to enable safety and efficacy evaluation in clinical VCA.

Permeation studies were performed with a solution of MPA (10 mg/ml). *In vitro* release and permeation studies were performed for different semisolid formulations (Aladerm, Lipoderm, emollient, and VersaBase) of MPA (1% w/w) using a Franz Diffusion Cell System (FDCS). *In vivo* pharmacokinetic characterization of MPA release from Lipoderm was performed in rats.

MPA in solution exhibited a steady state flux (3.8 ± 0.1 µg/cm^2^/h) and permeability (1.1 × 10^−7^ ± 3.2 × 10^−9^ cm/s). MPA in Lipoderm exhibited a steady state flux of 1.12 ± 0.24 µg/cm^2^/h, and permeability of 6.2 × 10^−09^ ± 1.3 × 10^−9^ cm/s across the biomimetic membrane. The cumulative release of MPA from Lipoderm, showed a linear single-phase profile with a R^2^ of 0.969. *In vivo* studies with MPA in Lipoderm showed markedly higher local tissue MPA levels and lower systemic MPA exposure as compared to values obtained after intravenous delivery of the same dose of drug (*p* < 0.05).

We successfully developed for the first time, a topical formulation of MPA in Lipoderm with optimal *in vitro*/*in vivo* permeability characteristics and no undesirable local or systemic adverse effects *in vivo*. Our study provides key preliminary groundwork for translational efficacy studies of topical MPA in pre-clinical large animal VCA models and for effectiveness evaluation in patients receiving VCA.

## Introduction

Over the past two decades, vascularized composite allotransplantation (VCA) has restored functional, psychosocial and quality of life outcomes in more than 250 patients suffering from devastating, unreconstructable extremity, craniofacial, genitourinary or other tissue defects. (1, 2)

The skin component of VCA (unlike in other solid organs), is touted to be the most immunogenic component and offers unique opportunities for graft monitoring (clinicopathologic correlation of rejection) and graft access for management (site-specific therapies). (3, 4) Conceivably, site-specific graft immunosuppression could reduce systemic exposure and global collateral or end-organ adverse effects of chronic systemic immunosuppression, which remains the “state of the art” in VCA. (5–8)

Currently, tacrolimus (TAC) (Protopic™ ointment 0.1%, 0.03%, Astellas), and clobetasol (Temovate ® ointment, cream 0.05%, GlaxoSmithKline), are FDA approved for topical use in certain dermatological conditions. (9–13) These topical immunosuppressive drugs have been used in VCA, off-label, to treat acute rejection *pro re nata* (PRN). (14) Despite their efficacy, these formulations are associated with undesirable local side effects. Topical TAC can cause local skin irritation and erythema associated with burning sensation, itching and pruritus. Importantly, the greasy formulation reduces medication adherence in patients (15–17) Topical clobetasol is associated with skin thinning or atrophy due to the impairment of collagen synthesis. (18–20)

With the exception of tacrolimus or clobetasol, there are no commercially available topical formulations for other widely used systemic immunosuppressive drugs such as mycophenolic acid (MPA), sirolimus (rapamycin, RAPA), and everolimus. It is therefore imperative to investigate the feasibility of developing alternative topical immunosuppressive drug formulations with independent or synergistic efficacy and safety profiles. (21) Developing an optimal topical formulation of MPA addresses this timely clinical need in VCA.

MPA is the active form of mycophenolate mofetil (MMF), which is an immunosuppressive drug used in solid organ transplantation (SOT). Unlike calcineurin inhibitors like cyclosporine and tacrolimus, MMF is not associated with nephrotoxicity or hepatotoxicity. (22–26) Over the past two decades, MMF has been used in triple therapy regimens in combination with TAC and prednisone in SOT (27–29) or VCA. (30–32) It has also been used in dual therapy in combination with RAPA in VCA. (33, 34)

MPA is available commercially in an enteric-coated form as mycophenolate sodium (Myfortic ®, Novartis) or the ester prodrug MMF (CellCept®, Roche), (8, 9). *In vivo*, the prodrug MMF is converted by hydrolysis to MPA (10, 11). This conversion occurs in blood, liver, kidneys and to a small extent in skin (12). MPA is metabolized by glucuronyl transferase to form MPA glucuronide (MPAG), which is an inactive metabolite that is excreted in the urine and bile. (35) In addition to its immunosuppressive effects, MPA has antibacterial, antifungal, antiviral, and antitumor properties. (36–38) MPA exerts its effects on T and B cells by reversible inhibition of inosine monophosphate dehydrogenase (IMPDH), an enzyme essential in the *de-novo*-synthesis of guanosine nucleotides required for DNA and RNA synthesis. (39, 40) Despite its therapeutic efficacy, systemic use of MPA/MMF has been associated with undesirable gastrointestinal (nausea, diarrhea, abdominal cramps, constipation, vomiting and anorexia) and genitourinary adverse effects (urgency, frequency, dysuria, hematuria and sterile pyuria). (41–43)

Topical drug administration of MPA in VCA could facilitate minimization of overall number of drugs, dosing, frequency and duration of systemic immunosuppression while improving its anti-rejection efficacy and effectiveness in graft survival. (3) Furthermore, such strategies could allow graft targeted delivery with predominantly localized action, reduced risk of systemic side effects, avoidance of first pass intestinal/liver metabolism, ability to administer multiple drugs in combination and potential for minimization of drug-drug interactions. (4–6, 21)

For efficacy, any transdermal drug formulation and delivery must first consider the challenging barrier of the stratum corneum in the skin. (44–47) Drugs with low molecular weight (≤500 g/mole), and high lipid solubility offer superior skin penetration and are suitable candidates for topical administration. (48, 49) The goal of this study was to prepare a topical formulation of MPA with optimal *in vitro*/in vivo characteristics such as release, permeation, and tissue bioavailability for further safety, efficacy and feasibility evaluation in clinical VCA.

## Materials, Animals, and Methods

### Chemicals and Reagents

MPA powder was obtained from Sigma–Aldrich (St. Louis, MO, USA). Cremophor EL (polyethoxylated castor oil, Kolliphor EL®, BASF, Germany), and propylene glycol USP were obtained from Sigma–Aldrich (St. Louis, MO, USA). Lipoderm, Aladerm, Versabase and Emollient cream were manufactured by PCCA (Professional Compounding Centers of America). Semisolid formulations of MPA Aladerm, MPA Lipoderm, MPA mollient, and MPA VersaBase were compounded by Hieber’s pharmacy (Pittsburgh, PA, USA). Spectra/pro RC membrane discs, molecular weight cut off (MWCO): 6–8000 Dalton, thickness 0.002 inches, diameter 33 mm, were purchased from spectrum chemicals ® (Rancho Dominguez, CA, USA). All the solvents were HPLC and MS grade and were obtained from Fisher Scientific (Pittsburgh, PA, USA). Sigmacote® siliconizing reagent for glass and other surfaces was obtained from Sigma–Aldrich (St. Louis, MO, USA).

### Animals

All experiments were performed in accordance with a protocol independently reviewed and approved by the University of Pittsburgh Institutional Animal Care and Use Committee (IACUC). Animals (inbred Male Lewis rats aged 8 to 10 weeks, weighing about 200 to 250 g, Charles River Laboratories, Horsham, PA), were housed in a specific pathogen-free barrier facility and maintained in accordance with IACUC guidelines. All procedures were in compliance with American Association for the Accreditation of Laboratory Animal Care (AALAC) recommendations and the principles set forth in the National Institute of Health Publication 80–23, Guide for the Care and Use of Laboratory Animals and the Animal Welfare Act of 1966, as amended. Rats were housed individually and in plastic Elizabethan collars to prevent oral ingestion of the topical formulations, and to prevent access of animal to the application site.

### Methods

#### Assessment of Partition Coefficient of MPA in Octanol/Water

Partition coefficient of MPA was experimentally measured to evaluate partitioning ability of MPA. 0.5–1 mg of drug powder was put in 2 mL tubes and sealed. One ml of 1-octanol and 1 mL of potassium phosphate buffer (pH = 7.4) was added to the drug powder. After vortexing for 5 min and ultra-sonication at 25°C for 15 min, samples were centrifuged for 5 min at 13,400 rpm to facilitate phase separation, and allowed to stand for 1 h. The octanol was separated from the aqueous phase. Samples were diluted with acetonitrile and analyzed by high-performance liquid chromatography. The partition coefficient (log P_o/w_) as a measure of lipophilicity was calculated as follows: $log\;P_{o/w}\;=\;log\;\left(C_{0}/C_{w}\right)$, C_0_ and C_w_ are the concentrations of MPA in the octanol and water phase, respectively as per standard methods.

### Preparation and Characterization of Semisolid Formulations

Semisolid formulations for MPA were compounded at Hieber’s pharmacy (Pittsburgh, PA, USA) using the following ingredients: MPA (Active ingredient), Propylene Glycol USP (wetting/solubilizing agent), and water, Isopropyl Myristate, Phospholipids, Cetearyl Alcohol Triticum Vulgare (Wheat) Germ Oil, Cetyl alcohol, Stearyl alcohol, Ceteareth-20, Caprylic/Capric, Triglycerides, Glycerin, Dimethicone C13–14 Isoparaffin, Laureth-7, Xanthan Gum, Magnesium Aluminum Silicate Polyacrylamide, Disodium EDTA, BHT, Phenoxyethanol, Methylchloroisothiazolinone, and Methylisothiazolinone (Base/excipients). The prepared formulation was grossly examined followed by light microscopy for appearance, color, and aggregates or lumps. Texture and consistency was optimized to eliminate grittiness. All formulations were stored at 4°C.

### Determination of in Vitro Release and Permeation (Penetration and Diffusion) of MPA in Solution and Semisolid Formulation

Permeation (partitioning and diffusion) of MPA in solution and four different semisolid formulations was simulated *ex vivo* in a Franz Diffusion Cell System (FDCS) usinga biomimetic membrane similar to human skin. The donor compartment was separated from the recipient compartment by a pre-hydrated biomimetic Spectra/Por® RC Membrane (0.002 inches thickness, 33 mm diameter, 0.45 µm pore size, MWCO: 6–8,000). (50) The effective diffusion area was 1.77 cm^2^. MPA in solution was prepared in a combination of cremophor 15% (Kolliphor EL®, BASF, Germany), ethanol 10%, and deionized water and the donor compartment was loaded with 400 µl of MPA (10 mg/ml) solution. Similarly, each semisolid formulation with MPA (0.5 g) was applied in the donor compartment. The aqueous recipient medium was magnetically stirred and maintained at 32–0.1°C on a hot plate. Samples were collected from recipient compartment at 0, 1, 2, 4, 6 and 24 h and replaced with the same volume of fresh recipient solution. Samples were analyzed by a validated HPLC assay developed in our laboratory. (51) All experiments were performed in triplicates and cumulative drug diffusion was calculated over a period of 24 h.

### In Vivo Evaluation of Topical MPA in Rats

Naïve Lewis rats received either a single topical dose of MPA in Lipoderm (1%, 16.6 mg/kg), [*n* = 6], or IV bolus dose (10 mg/kg), [*n* = 8]. The topical formulation was applied on the right or left hind limb of the rat. Tail vein blood sampling was performed at 0.08, 0.16, 0.25, 0.5, 1, 1.5, 2, 3, 4, 6, 8, 12 and 24 h. Blood samples were centrifuged at 2100 ± 100 rpm for 10 min, at room temperature and the plasma was separated and stored frozen at −80 C until analysis. Rats were euthanized after 24 h following MPA treatment and tissues [skin, muscle, and draining lymph nodes (DLN)] were collected from both the application limb and contralateral limb for drug level measurement. Biopsies from skin and muscle were collected at 2, 6, 12, 24 h post dose administration for measuring drug concentration. The skin site was wiped with 50% ethanol prior to tissue sampling for MPA. Plasma and tissue levels of MPA were analyzed by modification of a prior published HPLC-MS/MS methodology (51).

### Pharmacokinetic Analysis

Pharmacokinetic parameters after topical/systemic administration of MPA were obtained using Graph pad prism 6 and Winnonlin 6. Peak plasma level (C_max_), and trough plasma level (C_trough_) were observed values. The area under the plasma concentration vs. time curve (AUC_0–α_) for MPA was determined by the linear trapezoidal rule. Clearance (CL) was calculated as $\frac{Dose}{AUC_{0-\alpha }}$.

The absolute bioavailability of MPA after topical administration was obtained as:

$$F\;=\;\frac{\left({AUC}_{0-\alpha }\;topical\right)}{\left(Dose_{topical}\right)}\times \frac{Dose\;i.v.}{AUC_{0-\alpha }\;i.v.}$$

### Assay Protocols

#### Quantification of MPA in Diffusion Medium by High Pressure Liquid Chromatography (HPLC)

Five hundred microliters of methanol were added to 50 µl of sample solution (MPA in medium). Samples were centrifuged for 3 min at 13,000 rpm after vortexing for 2 min at 3,000 rpm. Supernatants (50 µl) were analyzed with an HPLC protocol developed and validated for determination of MPA in a medium made up from a mixture of cremophor 15%: ethanol 10%: water. Separation was performed by a reversed phase SYMMETRY C18 column (100 Å, 5 µm, 4.6 × 250 mm) using Water Alliance System 2,695–2,998 with UV detection at 254 nm. Isocratic elution was performed with a mobile phase consisting of 30% water, 70% methanol, 0.1% formic acid (pH = 3), flow rate of 1 ml/min, injection volume 50 µl, column temperature 37°C. MPA had a retention time of 4.2 min. The method was selective and reproducible in the range of 0.25–15 µg/ml with r^2^ of .9996. The lower limit of quantification (LLQ) was 0.25 µg/ml. The intraday and interday CV% at 0.5, 5 and 10 µg/ml were less than 10% (*n* = 3).

### Quantification of MPA in Plasma by HPLC-Tandem Mass Spectrometry (HPLC-MS/MS)

Acetonitrile (500 µl extracting solvent) containing MPA-D3 (internal standard, 250 ng/L) was added to 50 µl of sample solution (MPA in plasma), followed by 200 µl zinc sulfate (0.1M). Samples were centrifuged for 3 min at 13,000 rpm after vortexing for 2 min, and supernatants (20 µl) were tested using a simple and reproducible HPLC-MS/MS method developed and validated for determination of MPA in plasma. Separation was performed by reversed phase HPLC-MS/MS using a Waters Atlantis dC18 column (100 Å, 5 µm, 2.1 × 20 mm). Gradient elution was performed with a mobile phase consisting of solvent A: 95% water, 5% methanol, 0.1% formic acid and 2 mM ammonium acetate, and solvent B: 100% methanol, 0.1% formic acid and 2 mM ammonium acetate with flow rate of 0.4 ml/min, injection volume 20 µl, and column temperature 40°C. MPA was quantitated using internal standard MPA-D3 by a positive electro spray ionization mode using multiple reactions monitoring. MPA had a retention time of 7 min. The parent to product mass transitions (m/z) for MPA was 338.2→207.2, and MPA-D3 was 341.20→210.20. The method showed an acceptable linearity in the range of 0.3–25 µg/ml with r^2^ of 0.9996. The lower limit of quantification (LLQ) was 0.3 µg/ml. The intraday and interday CV% at 1, 5 and 10 µg/ml were less than 10% (*n* = 3). (51)

### Quantification of MPA in Tissues by Homogenization and Drug Extraction

The skin sites for tissue sampling were first wiped down three times with ethanol-soaked gauze to remove residual topical formulation on the surface. Skin, muscle, and DLN samples were frozen with liquid nitrogen. A BioPulverizer (BioSpec Products, Inc.) was used to pulverize/fragment tissue samples into powder form, 0.1 g of which was homogenized with methanol using a Mini-BeadBeater-1 (Cole-Parmer North America). Homogenate was left over night in a sonicator to allow for complete extraction of the drug from tissues. centrifuged at. Supernatants were collected by centrifugation of extracted tissue homogenates (2,100 ± 100 rpm for 10 min) and evaporated in a sample concentrator. Drug residue was then reconstituted with plasma and tissue drug concentration was calculated as µg/g of tissue weight.

### Statistical Analysis and Data Interpretation

Statistical analysis was performed using parametric tests as the data was compatible with normality assumptions (numerical, linear relationships, distributions had normal shape). Student *t* test was used for two groups and ANOVA was used when one independent variable with greater than two conditions or treatments and outcomes was evaluated and compared. All experimental results were expressed as the mean ± SD deviation (SD). A *P* value < 0.05 was considered statistically significant.

## Results

### MPA Permeability and Drug Characteristics

The molecular weight of MPA is 320.34 g/mole. The partition coefficient (log P) for MPA was experimentally measured as 3.5 ± 0.1, while the predicted value was 3.8. The flux (Jss) value was 3,842 ± 115 µg/cm^2^/h, while the permeability coefficient (Kp) was 9.8 × 10^−8^ cm/s.

### Physical Characterization of Semisolid Formulation of MPA

The topical formulation demonstrated good homogeneity with absence of aggregates or clumping and excellent texture with no grittiness. The pH of the formulation was 5.4 ± 0.5 which is very comparable to the pH of native skin. (52)

### In Vitro Permeation of MPA in Solution

**Figure 1:**
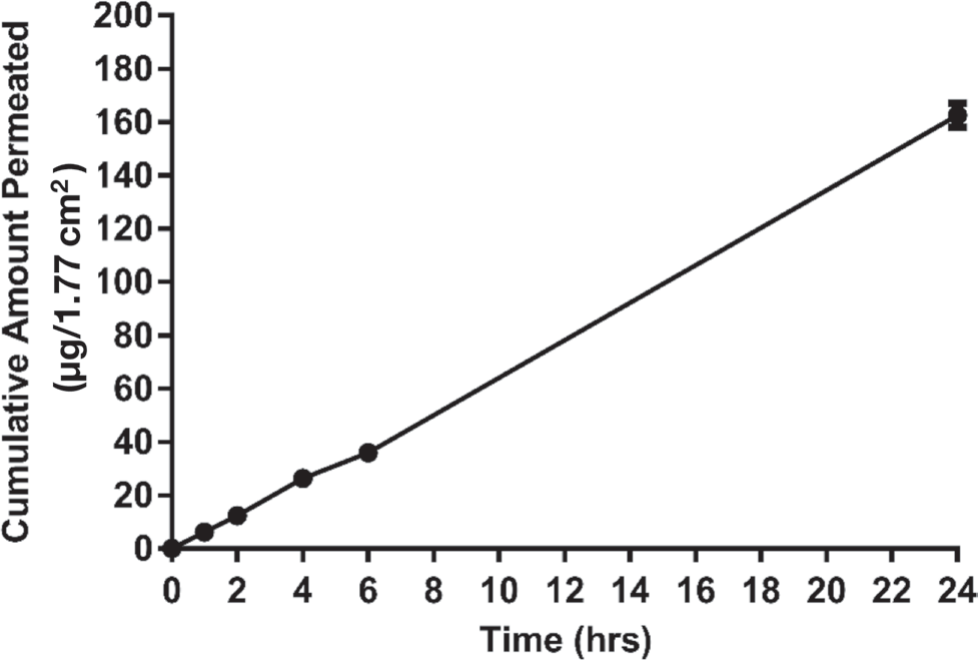
Cumulative amount (Mean ± SD) of MPA permeated across a synthetic membrane from MPA solution vs. Time (n = 3). The cumulative release amount of MPA in solution, plotted against the time, showed a linear profile with R^2^ of 0.969. Plateau state was not reached in 24 h. Highpercentage of MPA dose (67.8%) was released from the donor chamber over 24 h, and only small percentage (7%) permeated into the receptor chamber.

The cumulative amount of drug permeated per unit area 1.77 cm^2^ from the solution of MPA across membrane over 24 h is shown in Figure 1. MPA permeation across the barrier membrane in FDCS followed the Fick’s Law of passive diffusion, J = K.C_v_/h, where J is flux, Cv is permeant concentration in vehicle, h is membrane thickness and K is the partition constant of the permeant between the membrane and vehicle.MPA in solution exhibited a steady state flux J_ss_ (3.8 ± 0.1 µg/cm^2^/h) with a 3% coefficient of variation (CV) of flux. Permeability Kp of MPA across the membrane was 1.1 × 10^−7^ ± 3.2 × 10^−9^ cm/s. A cumulative release amount of MPA in solution, plotted against the time, showed a linear profile with R^2^ of 0.969. Total amount of MPA permeated over 24 h was 163 ± 4.6 µg. 67.8% of the loaded MPA dose was released from the donor chamber over 24 h. 7% of the loaded MPA dose permeated over 24 h into the receptor chamber.

### In Vitro Release Profile of MPA in Semisolid Formulation

**Figure 2:**
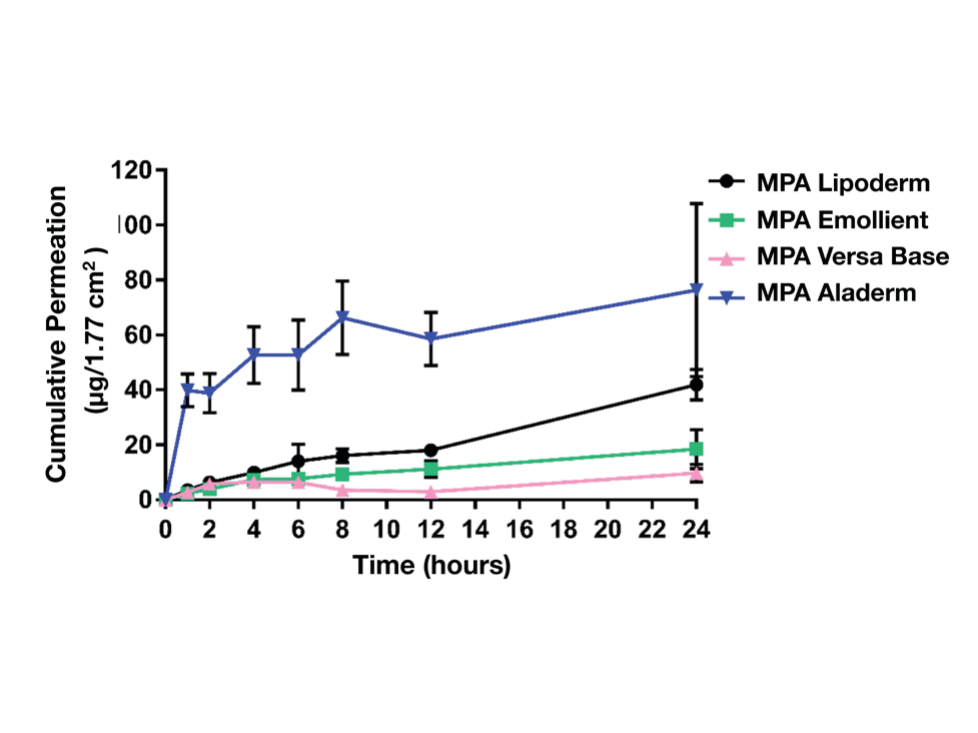
Cumulative amount (Mean ± SD) of MPA permeated per 1.77 cm^2^ from semisolid formulations vs. Time (n = 3). Formulations tested include Aladerm, Lipoderm, VersaBase and emollient base. Of the four semisolid formulations tested, MPA in Aladerm exhibited a high initial release rate (2.5 × 10^−05^ ± 1.6 × 10^−05^ cm/h) followed by MPA in Lipoderm (2.2 × 10^−05^ ± 4.7 × 10^−06^ cm/h). MPA in emollient and MPA in VersaBase exhibited lower initial release rates (8.1 × 10^−06^ ± 3.4 × 10^−06^ and 2.8 × 10^−06^ ± 1.5 × 10^−06^ cm/h). The cumulative release of MPA from Lipoderm, showed a linear single-phase profile over time with R^2^. Most of MPA was retained in the membrane and only small amount of MPA slowly permeated into the receptor chamber (42 ± 5 µg).

The total amount of MPA permeated from four different formulations into the receptor chamber over 24 h from the plots is presented in Figure 2 and shown in Table 1. MPA in Aladerm exhibited a high initial release rate (2.5 × 10^−05^ ± 1.6 × 10^−05^ cm/h) followed by MPA in Lipoderm (2.2 × 10^−05^ ± 4.7 × 10^−06^ cm/h). MPA in emollient and MPA in VersaBase exhibited a lower initial release rate (2.8 × 10^−06^ ± 1.5 × 10^−06^ cm/h).

**Table 1:** Mean total amount of MPA (Mean ± SD) permeated from four different formulations into the receptorchamber over 24 h (Mean ± SD, n = 3)

**Formulations**	**Total Permeated Amount over 24 h (µg)**
**MPA in Aladerm**	76.3 ± 31.5
**MPA in Lipoderm**	42 ± 5
**MPA in Emollient**	18.5 ± 7
**MPA in Versa Base**	10 ± 3.3

MPA in Lipoderm exhibited steady state flux (1.12 ± 0.24 µg/cm^2^/hr) and permeability (6.2 × 10^−09^ ± 1.3 × 10^−09^ cm/s) across the biomimetic membrane in linear fashion. The cumulative release of MPA from Lipoderm, showed a linear single-phase profile (Gradual and sustained over prolonged period of time). (53) A high percentage of the loaded MPA dose was released, while only a small fraction of MPA permeated into the receptor chamber. Several models were tested with the data to select the model with the best fit for the total drug release kinetic profile. The Higuchi’s model was a good fit for our experimental data as it is one of the release kinetic models that describes the released amounts of compounds as a function of square root of time. The diffusion coefficient of MPA in the membrane was 2.8 × 10^−05^.

### In Vivo Profile of MPA After Topical Administration

**Figure 3:**
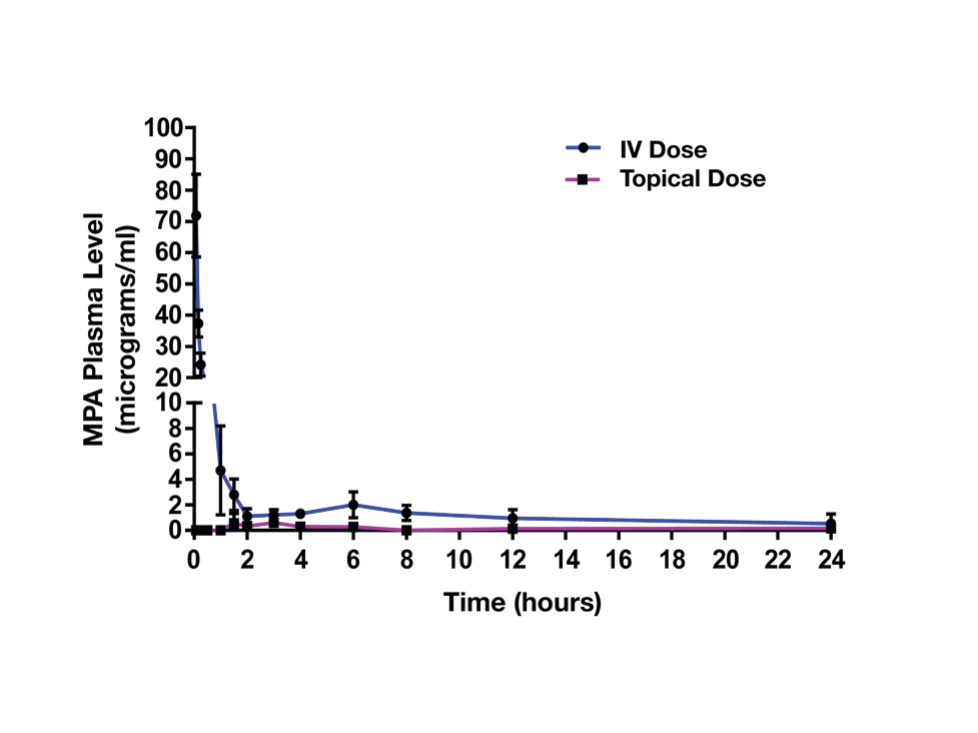
Plasma MPA concentrations (Mean +/- SD) following an IV bolus dose (10 mg/kg, n = 8) and/or topical dose (16.6 mg/kg, n = 6) in naïve Lewis rats. Following IV bolus administration, peak plasma concentration was reached at 0.08 h with 71.8 ± 28 µg/ml, and then concentrations quickly declined over time to reach low values (0.5 ± 0.7 µg/ml) at 24 h. Following topical administration, the peak MPA concentration was appeared between 3 and 4 h with 0.6 ± 0.3 µg/ml, concentrations were very low, and undetectable (<0.3 µg/ml) at 24 h. Peak levels C _max_ and total systemic drug exposure AUC_0-α_ after topical administration was significantly lower than the values observed after IV bolus administration (****p < 0.0001).

**Figure 4:**
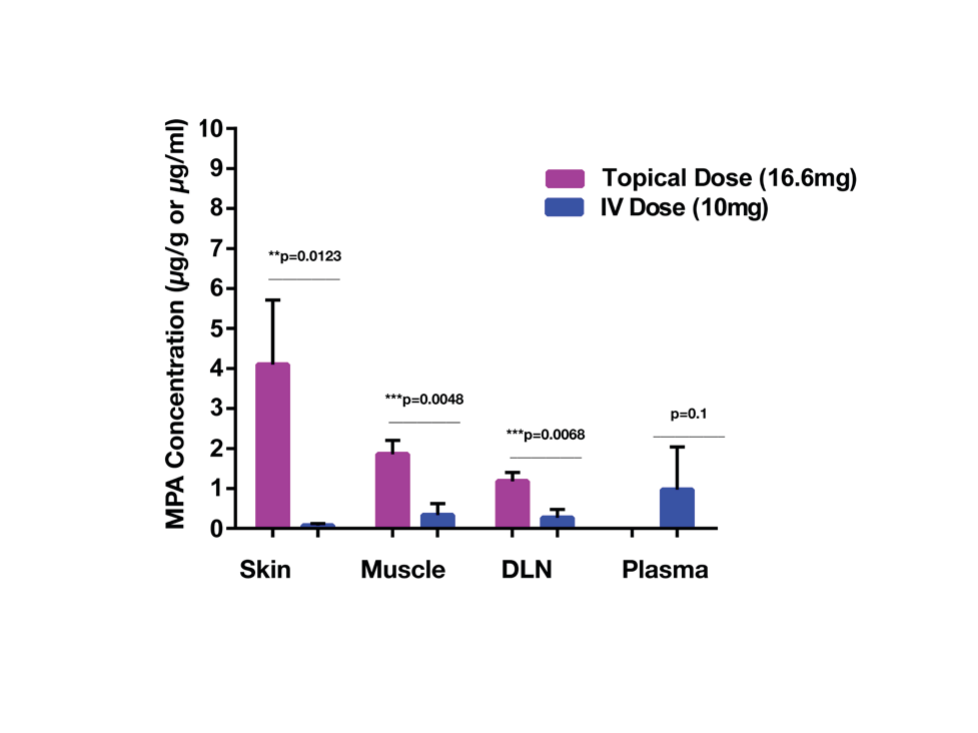
MPA concentrations (Mean ± SD.) in plasma and local tissues (skin, muscle, DLNs) following a topical (16.6 mg/kg) or IV dose (10 mg/kg) in rats (n = 3). MPA concentrations in tissues were much higher after topical administration as compared to IV administration (*p* < 0.05), which indicates predominant localization of drug delivery at the site of application. MPA concentrations in skin, muscle, and DLNs after topical administration vs. IV administration (**p = 0.0123, ***p = 0.0048, ***p = 0.0068).

**Figure 5:**
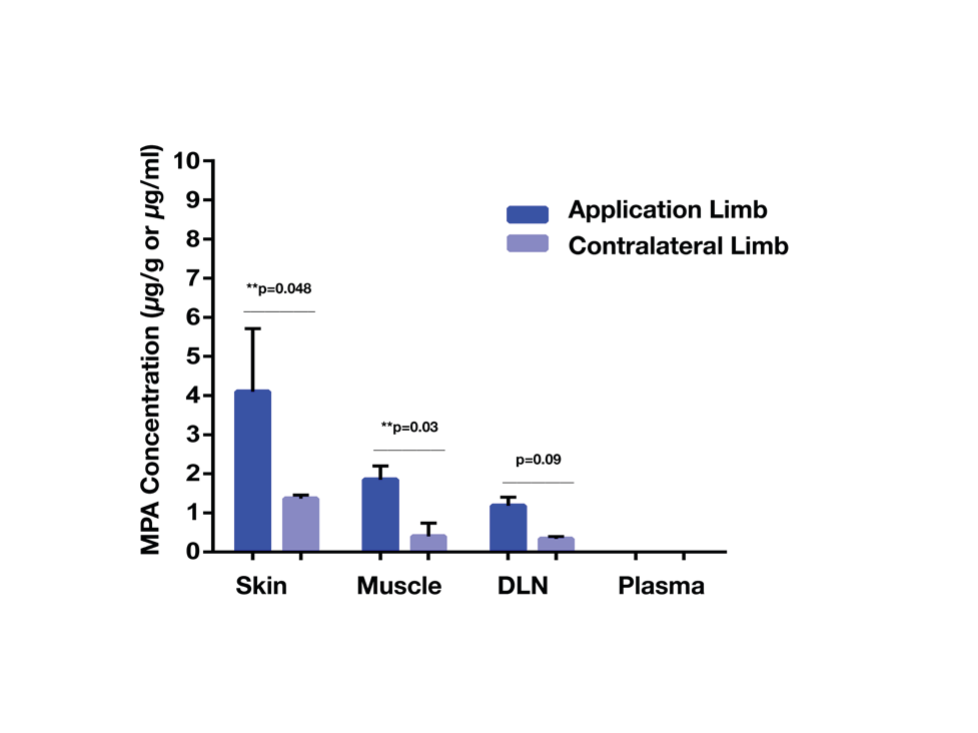
MPA concentrations (Mean ± SD) in plasma and tissues (application limb and contralateral limb) following a topical dose (16.6 mg/kg) in rats (*n* = 3). MPA concentrations in skin, muscle, draining lymph nodes (DLN) collected from the application limb following a topical dose in rats are much higher than MPA concentrations in the other contralateral tissues (*p* < 0.05). MPA concentrations in skin, muscle, and DLN collected from application limbs vs. contralateral limbs (*p = 0.04, **p = 0.03, *p* = 0.09).

**Figure 6:**
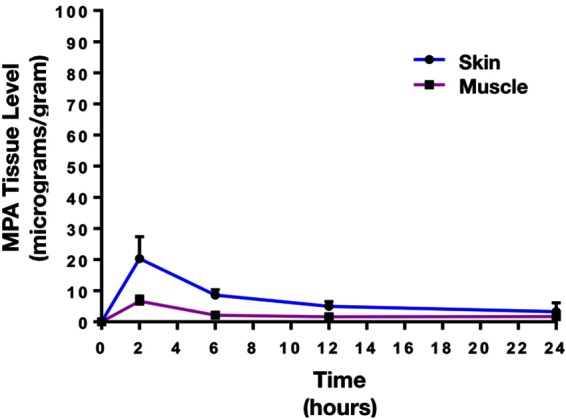
Time course of MPA concentrations (Mean ± SD) in skin and muscle following a topical dose (16.6 mg/kg) in rats (n = 3). Peak plasma concentrations of MPA in skin and muscle were reached 2 h post-topical dose administration (20.4 ± 7 and 6.6 ± 1.6 µg/g), then concentrations gradually declined over time to reach low values at 24 h post topical dose administration (3.2 ± 2.8 and 1.7 ± 1.5 µg/g). Peak levels C _max_ and total systemic drug exposure AUC_0-α_ in the skin were higher than the values observed in the muscle (*p* < 0.01). MPA was cleared from the skin in a lower rate than the muscle (32 ± 1.2 vs. 68.6±39.7 l/h).

The mean plasma concentration-time profile of MPA after single dose topical and/or IV administration is presented in Figure 3. Following IV bolus administration, the MPA concentrations were very high initially (71.8 ± 28 µg/ml), with concentrations declining quickly thereafter over time to reach low values (0.5 ± 0.7 µg/ml) at 24 h. Following topical administration, peak MPA concentrations (0.6 ± 0.3 µg/ml) were reached between 3 and 4 h. Concentrations of MPA were very low, or undetectable (<0.3 µg/ml) at 24 h. The pharmacokinetic parameters of MPA such as systemic exposure (AUC _0–∞_), maximum drug levels (C _max_), trough drug levels (C _trough_), and bioavailability after topical and/or IV dose in rats is shown in Table 2. The C_max_ and AUC_0–α_ values were much lower after topical administration as compared to systemic administration of MPA (****p < 0.0001). MPA concentrations in plasma and local tissues including skin, muscle, lymph nodes following a topical dose and/or IV dose in rats is presented in Figure 4 and shown in Table 3. MPA concentrations in limb tissues were much higher after topical administration as compared to IV administration (*p* < 0.05), which indicates the localization of drug delivery to the site of application. MPA concentrations in skin, muscle, and DLN after topical administration vs. IV administration (**p = 0.0123, ***p = 0.0048, ***p = 0.0068). MPA concentrations in plasma and tissues collected from the application limb following a topical dose in rats is much higher than MPA concentrations in the other contralateral limb tissues (*p* < 0.05) (Figure 5). MPA concentrations in skin, muscle, and DLN collected from application limbs vs. contralateral limbs (*p = 0.04, **p = 0.03, *p* = 0.09). Time course of MPA concentrations in skin and muscle at 2, 6, 12, and 24 h following a topical dose (16.6 mg/kg) in rats is presented in Figure 6**.** Pharmacokinetic parameters of MPA in the skin and muscle following a topical dose (16.6 mg/kg) in rats (*n* = 3) is shown in Table 4. Peak plasma concentrations of MPA in skin and muscle were achieved 2 h after topical administration (20.4 ± 7 and 6.6 ± 1.6 µg/g respectively)/Thereafter, MPA concentrations declined over time to reach low values at 24 h post topical dose administration (3.2 ± 2.8 and 1.7 ± 1.5 µg/g respectively). Peak levels C _max_ and total systemic drug exposure AUC_0–α_ in the skin were higher than the values observed in the muscle (*p* = 0.01). MPA was cleared from the skin at a lower rate than from muscle (32 ± 1.2 vs. 68.6 ± 39.7 l/h). During the first 2 h, MPA concentration in the skin increased at a faster rate than in the muscle, with slope = 10.14 for skin and slope = 3.33 in the muscle. After 2 h, the concentrations began to decrease in both tissues.

**Table 2:** Plasma pharmacokinetic parameters of MPA (mean + SD) in rats after IV bolus (10 mg/kg, n = 8) or topicaldose (16.6 mg/kg, n = 6).

**Route of administration**	**Dose (mg/kg)**	**AUC**_**0–α **_**(µg.h/ml)**	**C **_**max **_**(µg/ml)**	**C **_**trough**_** (µg/ml)**	**CL/F (ml/h)**	**Bioavailability (%)**
**Intravenous administration**	10	34.4 ± 5.8	71.8 ± 28	0.5 ± 0.7	78.5 ± 16.8	100
**Topical administration**	16.6	3.8 ± 1.1	0.6 ± 0.3	ND	1323 ± 355	6

**Table 3:** MPA concentrations (Mean + SD) inskin, muscle and draining lymph node (DLN) tissue and plasmafollowing IV bolus (10 mg/kg, n = 3) or topical dose (16.6 mg/kg, n = 3)

**MPA Concentration**	**IV Delivery**	**Topical Delivery**
**Skin**	0.06 ± 0.05	4 ± 1.6
**Muscle**	0.3 ± 0.2	1.85 ± 0.3
**DLNs**	0.26 ± 0.2	1.2 ± 0.2
**Plasma**	0.7 ± 0.1	0.2 ± 0.2
**Skin-Plasma ratio**	0.085	20
**Muscle-Plasma ratio**	0.4	3.2
**DLN-Plasma ratio**	0.37	6

**Table 4:** Pharmacokineticparameters of MPA (Mean + SD) in the skin and muscle following atopical dose (16.6 mg/kg) in rats (n = 3)

**Tissue Type**	**AUC**_**0–α**_** (µg.h/ml)**	**C **_**max**_** (µg/ml)**	**C **_**trough**_** (µg/ml)**
**Skin**	168.5 ± 19	20 ± 7	3.2 ± 2.8
**Muscle**	55 ± 9	6.6 ± 1.6	1.7 ± 1.5

## Discussion

Upon topical application, MMF undergoes only limited or unpredictable metabolism to the active form, MPA in the skin. This is because, skin esterase levels are variable depending on the location of application. (54) Our study takes the logical approach to develop a topical formulation with MPA rather than MMF. Topical administration of MPA, the “active drug,” via the skin or mucosa in VCA circumvents the confounder of skin esterase activity and could improve effectiveness by predominantly concentrating drug levels in the graft, decreasing systemic exposure, and consequentially, off-target effects.

The use of topical MPA in VCA could also be synergistic with topical TAC or other agents. It could help minimize the need for systemic MMF, TAC or corticosteroids for the prevention/treatment of allograft rejection, and augment anti-rejection efficacy and medication adherence in patients, while lowering risk of systemic adverse effects.

Topical delivery of MMF has been attempted in dermatology applications such as psoriasis, vitiligo, atopic dermatitis or allergic contact dermatitis with varying results. (55–58) Although MMF is relatively lipophilic, skin permeation may be challenged by the stratum corneum (SC) whichis a natural barrier that limits systemic drug absorption and exposure. A thickened SC is the cause of treatment failures in psoriasis with topical MMF (59) requiring the need for penetration enhancers such as eucalyptol (EUL) and N-methyl-2-pyrrolidone (NMP). (60) However, these enhancers inherently  cause skin irritation. (61)

To date, no study has compared the use of different formulation bases for topical delivery of MMF or MPA either in dermatology or VCA applications. Also, to our knowledge, a formal analysis of the pharmacokinetics and bioavailability of the active form of MPA, especially across the skin barrier has not been reported.

Ideally, topical delivery should be tested across a skin barrier. Excised human skin is considered the gold standard model for *in vitro* drug permeation and penetration assessment. (62) However, large variations are common across human skin explants due to differences in age, gender, race and anatomical donor site. (46) On the other hand, animal skins from pigs (porcine ear), guinea pigs, hairless mice or snakes (ecdysial skin) have been used as predictive model systems for *in vivo* human penetration/permeation oftopical agents. (63, 64) But, there is significant intra- and inter-individual variation between animal and human skin, when skin characteristics, such as thickness of skin (especially SC), lipid content, density of hair follicles, and esterase enzyme activity in each model are compared. (65) Studies have shown that the skin of rodents, such as hairless rats and hairless mice, are more permeable than human skin using drugs/agents with different physicochemical properties. (66) Some of the critical parameters that cause such variability in permeation/penetration proﬁles in animal or human skin are effects of storage and freezing (use of cryopreserving agents such as 10% glycerol) that can cause alterations in skin hydration or electrical resistance. (66–68) This can alter permeability and the lag time of hydrophobic drugs such as MPA as tested in this study. Finally, there is no consensus on the use of an ideal cryoprotectant for skin preservation or the optimal storage time/conditionsfor frozen skin used for *in vitro* drug permeation/penetration studies. (69) To overcome these individual limitations with animal or human skin, and to optimize testing of the topical MPA delivery parameters, our study combined *in vitro* and *in vivo* evaluation of efficacy and safety of topical MPA for skin applications.

We first evaluated our formulations *in vitro* in a FDCS system across a regenerated cellulose dialysis membrane (SpectraPor® RC) that functions like a biomimetic skin barrier. Our choice of the FDCS system was based on its validated metrics (such as membrane parameters, cell dimensions, temperature, membrane treatment, stirring efficiency, sampling frequency).

MPA in solution exhibited a good steady state flux (CV 3%) and permeability (Kp 1.1 × 10^−7^ ± 3.2 × 10^−9^ cm/s) across the biomimetic membranein a linear fashion (Fick’s law), and saturation or plateau state was not reached in 24 h. During the Franz cell run, the saturated state of MPA in solution was maintained to keep the thermodynamic activity constant and sustain sink conditions via frequent sampling rates (every 15–30 min).

Careful correlation of characteristics such as permeability coefficient (*P*), diffusivity (*D*), and partition coefficient (*K*) for each of the four semisolid formulations tested were important considerations in our study. The highest initial release rate, mean steady state flux, permeability, and total permeation over 24 h were seen with MPA in Aladerm, followed by MPA in Lipoderm, MPA in emollient and MPA in VersaBase. MPA in Aladerm exhibited the highest diffusion and fast initial release (burst) and amount permeated to the receptor. This could indicate a propensity for rapid systemic absorption and high exposure with clinical use. Furthermore, Aladerm has a fluid texture that may lead to difficulty in application and maintaining the formulation on the skin for reliable absorption and efficacy. We thus excluded MPA in Aladerm from further testing. Similarly, we excluded MPA in emollient and VersaBase as choices for further development because of their thick and greasy nature, which challenges topical application (difficulty in washing, staining of clothes and reduced patient adherence). (70, 71)

Contrary to Aladerm, only a moderate amount of MPA permeated into the receptor chamber over 24 h with the Lipoderm formulation. The timeline and kinetics of drug diffusion and release were ideal with MPA in Lipoderm. This included gradual diffusion and sustained drug release which facilitates a prolonged local site-specific action of the drug. Also, the formulation was shelf stable (with no degradation or alterations in pH or composition) over 3 months of storage at 25°C. The pH of the formulation was close to natural skin pH (on average 4.7), (52) minimizing risk of irritation.

While a high percentage of MPA dose was released from MPA in solution, only small percentage permeated into the receptor chamber. However, in comparison to MPA in solution, we observed a lower total permeability of MPA from all semisolid formulations across the biomimetic membrane.Our *in vitro* data with both MPA in solution and semisolid formulation was robust and reproducible as our methodology and experimental conditions were kept constant throughout the FDCS runs.

Our *in vivo* results demonstrated that AUC_0–∞_, C _max_, and C _trough_ after topical delivery of MPA were markedly lower than the values obtained after systemic delivery of MPA. Low concentrations of MPAwere observed in skin and DLNs after 24 h with a single IV dose, indicating that multiple, high dose injections may be necessary to increase concentrations in these tissues. Conversely, the low systemic bioavailability of MPA as observed after topical administration was probably due to drug accumulation in the local tissues at the site of application. MPA concentrations in tissues (skin and muscle) collected from the application site after topical delivery were significantly higher than values observed after systemic administration.The SC barrier of the skin may also slow or limit the rate of systemic drug absorption and release into circulation. In fact, the skin component retained the most drug with topical application of MPA in Lipoderm when levels across skin, muscle, DLNs and plasma were compared (Figures 4, 5; Tables 3, 4). MPA concentrations in skin and muscle reached the highest values 2 h post-topical administration, which indicates relatively rapid uptake of the drug into the skin and muscle tissue. Drug levels gradually fell to reach low concentrations at 24 h post-topical dose administration due to drug metabolism or clearance into the systemic circulation. Drug concentration in tissues (skin, muscle, and DLNs) collected from the application site was significantly higher than drug concentrations in tissues collected from the contralateral site (Figure 5). This confirms that MPA predominantly localizes to the site of topical application with limited dispersion to other sites remote from the zone of topical delivery.

The high drug levels in DLNs may relate to the hydrophobic/lipophilic nature of MPA. It is known that lipophilic agents are preferentially taken up by the lymphatic system and the degree of uptake depends on factors such as particle size (size range 200–600 nm), surface charge, molecular weight, and hydrophobicity. (72, 73) DLNs are the initial site of allorecognition, and thereby localization of higher concentrations of MPA in these tissues could curb innate or adaptive immune responses in VCA tissues.

Taken together, MPA in Lipoderm exhibited the optimal profile as the formulation of choice for topical use in clinical VCA. Future *in vitro* studies are being planned to evaluate the long-term stability of this formulation to determine the shelf life and optimal storage conditions consistent with clinical use.Studies are also underway in small and large animal VCA, to establish the effectiveness of MPA in Lipoderm (with or without low dose systemic immunosuppression) in preventing/reversing acute skin rejection or chronic vascular rejection, and sustaining allograft survival without systemic toxicity. In addition to its use as a single agent in topical therapy for VCA, MPA can be combined with topical TAC or RAPA for synergistic efficacy on T cell responses. (42) In fact, topical therapy combining MPA and RAPA provides complementary inhibition of Th2-related cytokines (IL-4) and Th1-related cytokines (IFN-γ) in atopic dermatitis.(57) Optimization of topical MPA formulations could thus lead to effective combination topical immunosuppression protocols (+RAPA ± TAC) for site-specific therapies (±low dose systemic immunosuppression) in VCA to prevent AR or chronic rejection. Potentially, targeting distinct mechanistic pathways and molecular targets in the skin immune system with a combination of topically effective, site-specific immunosuppression may facilitate a permissive, immunomodulatory milieu that enables prolonged graft survival in VCA with minimization of systemic immunosuppression and long-term drug-related toxicity.

## Ethics Statement

This study was approved by University of Pittsburgh, the Institutional Animal Care and Use Committee (IACUC) (Protocol # 16027325).

## Author Contributions

Overall research idea, conceptual approach and hypothesis (VG), Experimental design (VG, RV, MS, MW), Protocol development and collection of data (FF, MW, JS, JP, WCZ, WZ); Manuscript drafting (FF), Extensive manuscript editing and rewriting (VG, RV), Additional input (MS, AS, LF, JP, JS); Analysis of data and interpretation of results (VG, RV, FF, MS, MW); Drug delivery design and development (RV, MW, FF). MW performed the in vitro experiments and initiated the study. FF performed the in vivo experiments, tissue processing for drug level measurements, analysis of data from in vitro and in vivo experiments, statistical analysis, and drafting of the manuscript. JS, VE and SO helped complete the in vivo experiments. WCZ performed the HPLC experiments and analysis of data. WZ helped with data analysis.

## Conflict of Interest Statement

The authors declare that the research was conducted in the absence of any commercial or financial relationships that could be construed as a potential conflict of interest.

The reviewer KA and handling Editor declared their shared affiliation, and the handling Editor states that the process nevertheless met the standards of a fair and objective review.

## References

[1] Gorantla VS Plock JA Davis MR Reconstructive Transplantation: Evolution, Experience, Ethics, and Emerging Concepts, Anesthesia and Perioperative Care for Organ Transplantation. New York, NY: Springer New York (2016). p. 539–52.

[2] Gorantla V Maldonado C Frank J Barker JH Composite Tissue Allotransplantation (CTA): Current status and future insights. Eur J Trauma (2001) 27(6):267–74. 10.1007/s00068-001-1152-1

[3] Schnider JT Weinstock M Plock JA Solari MG Venkataramanan R Zheng XX Site-specific immunosuppression in vascularized composite allotransplantation: prospects and potential. Clin Dev Immunol (2013) 2013(3):1–7. 10.1155/2013/495212 PMC3586464 23476677

[4] Solari MG Washington KM Sacks JM Hautz T Unadkat JV Horibe EK Daily topical tacrolimus therapy prevents skin rejection in a rodent hind limb allograft model. Plast Reconstr Surg (2009) 123(2 Suppl):17S–25. 10.1097/PRS.0b013e318191bcbd 19182660

[5] Lawson SD Fries CA Wang LC Gorantla VS Davis MR Locally administered immunomodulation for the maintenance of vascularized composite allotransplants. Plast Reconstr Surg (2015) 136(4 Suppl):78 10.1097/01.prs.0000472378.04620.fd

[6] Unadkat JV Schnider JT Feturi FG Tsuji W Bliley JM Venkataramanan R Single implantable FK506 disk prevents rejection in vascularized composite allotransplantation. Plast Reconstr Surg (2017) 139(2):403e–14. 10.1097/PRS.0000000000002951 28121868

[7] Schneeberger S Gorantla VS Hautz T Pulikkottil B Margreiter R Lee WP Immunosuppression and rejection in human hand transplantation. Transplant Proc (2009) 41(2):472–5. 10.1016/j.transproceed.2009.01.019 19328906

[8] Gharb BB Rampazzo A Altuntas SH Madajka M Cwykiel J Stratton J Effectiveness of topical immunosuppressants in prevention and treatment of rejection in face allotransplantation. Transplantation (2013) 95(10):1197–203. 10.1097/TP.0b013e31828bca61 23532181

[9] Funaro D Lovett A Leroux N Powell J A Double-Blind PJ A double-blind, randomized prospective study evaluating topical clobetasol propionate 0.05% versus topical tacrolimus 0.1% in patients with vulvar lichen sclerosus. J Am Acad Dermatol (2014) 71(1):84–91. 10.1016/j.jaad.2014.02.019 24704090

[10] Ho N Pope E Weinstein M Greenberg S Webster C Krafchik BR A double-blind, randomized, placebo-controlled trial of topical tacrolimus 0.1% vs. clobetasol propionate 0.05% in childhood vitiligo. Br J Dermatol (2011) 165(3):626–32. 10.1111/j.1365-2133.2011.10351.x 21457214

[11] Hettiarachchi P Hettiarachchi RM Jayasinghe RD Sitheeque M Comparison of topical tacrolimus and clobetasol in the management of symptomatic oral lichen planus: a double-blinded, randomized clinical trial in Sri Lanka. J Investig Clin Dent (2017) 8(4):e12237 10.1111/jicd.12237 27633647

[12] Madan V August PJ Chalmers RJ Efficacy of topical tacrolimus 0.3% in clobetasol propionate 0.05% ointment in therapy-resistant cutaneous lupus erythematosus: a cohort study. Clin Exp Dermatol (2010) 35(1):27–30. 10.1111/j.1365-2230.2009.03351.x 19549244

[13] Tzung TY Liu YS Chang HW Tacrolimus vs. clobetasol propionate in the treatment of facial cutaneous lupus erythematosus: a randomized, double-blind, bilateral comparison study. Br J Dermatol (2007) 156(1):191–2. 10.1111/j.1365-2133.2006.07595.x 17199600

[14] Kueckelhaus M Fischer S Seyda M Bueno EM Aycart MA Alhefzi M Vascularized composite allotransplantation: current standards and novel approaches to prevent acute rejection and chronic allograft deterioration. Transpl Int (2016) 29(6):655–62. 10.1111/tri.12652 26265179 PMC4785085

[15] Furue M Terao H Moroi Y Koga T Kubota Y Nakayama J Dosage and adverse effects of topical tacrolimus and steroids in daily management of atopic dermatitis. J Dermatol (2004) 31(4):277–83. 10.1111/j.1346-8138.2004.tb00673.x 15187322

[16] Yentzer B Clark A Williams L Sagransky M Adherence to topical tacrolimus 0.1% ointment in children with atopic dermatitis. J Am Acad Dermatol (2010) 62(3):AB46.

[17] Yang M-Y Jin H Shim W-H High rates of secondary non-adherence causes decreased efficacy of 0.1% topical tacrolimus in adult eczema patients: results from a multicenter clinical trial. J Dermatolog Treat (2017) 15:1–6. 10.1080/09546634.2017.1350256 28670943

[18] Furue M Terao H Rikihisa W Urabe K Kinukawa N Nose Y Clinical dose and adverse effects of topical steroids in daily management of atopic dermatitis. Br J Dermatol (2003) 148(1):128–33. 10.1046/j.1365-2133.2003.04934.x 12534606

[19] Shlivko IL Kamensky VA Donchenko EV Agrba P Morphological changes in skin of different phototypes under the action of topical corticosteroid therapy and tacrolimus. Skin Res Technol (2014) 20(2):136–40. 10.1111/srt.12095 23808907

[20] Lubach D Rath J Kietzmann M Steroid-Induced Dermal Thinning: Discontinuous Application of Clobetasol-17-Propionate Ointment. Dermatology (1992) 185(1):44–8. 10.1159/000247402 1638070

[21] Feturi F Okzus S Solari M Venkataramanan R Gorantla V The therapeutic efficacy of local delivery of a combination of tacrolimus and mycophenolic acid along with lower doses of systemic immunotherapy in prolongation of the composite tissue allograft survival with a minimum systemic toxicity. Transplantation (2014) 98:408 10.1097/00007890-201407151-01347

[22] Land W Vincenti F Toxicity-sparing protocols using mycophenolate mofetil in renal transplantation. Transplantation (2005) 80(2 Suppl):S221–34. 10.1097/01.tp.0000186386.13597.cb 16251855

[23] Schwarz C Oberbauer R Calcineurin inhibitor sparing in renal transplantation. Curr Opin Organ Transplant (2006) 11(6):632–6. 10.1097/MOT.0b013e328010c511

[24] Moore J Middleton L Cockwell P Adu D Ball S Little MA Calcineurin inhibitor sparing with mycophenolate in kidney transplantation: a systematic review and meta-analysis. Transplantation (2009) 87(4):591–605. 10.1097/TP.0b013e318195a421 19307799

[25] Goralczyk AD Bari N Abu-Ajaj W Lorf T Ramadori G Friede T Calcineurin inhibitor sparing with mycophenolate mofetil in liver transplantion: a systematic review of randomized controlled trials. Am J Transplant (2012) 12(10):2601–7. 10.1111/j.1600-6143.2012.04157.x 22813081

[26] Keunecke C Rothenpieler U Zanker B Schneeberger H Illner W-D Theodorakis J Mycophenolate mofetil monotherapy: an example of a safe nephrotoxicity/atherogenicity-free immunosuppressive maintenance regimen in a selected group of kidney-transplanted patients. Transplant Proc (2000) 32(1):6–8. 10.1016/S0041-1345(00)00808-3 10686310

[27] Offermann G Five-year results of renal transplantation on immunosuppressive triple therapy with mycophenolate mofetil. Clin Transplant (2003) 17(1):43–6. 10.1034/j.1399-0012.2003.02101.x 12588321

[28] Ana Luisa RP Manuel Alejandro MF Clinical Pharmacokinetics of Triple Immunosuppression Scheme in Kidney Transplant (Tacrolimus, Mycophenolate Mofetil and Corticosteroids). Understanding the Complexities of Kidney Transplantation. InTech (2011).

[29] Crespo JF Górriz JL Sancho A Ávila A Alcoy E Pallardó LM Triple therapy with mycophenolate mofetil, cyclosporine, and prednisone in renal transplantation. Transplant Proc (1999) 31(6):2261–2. 10.1016/S0041-1345(99)00329-2 10500568

[30] Breidenbach WC Gonzales NR Kaufman CL Klapheke M Tobin GR Gorantla VS Outcomes of the first 2 American hand transplants at 8 and 6 years posttransplant. J Hand Surg Am (2008) 33(7):1039–47. 10.1016/j.jhsa.2008.02.015 18762094

[31] Gorantla VS Breidenbach WC Hand Transplantation: The Louisville Experience, Transplantation of Composite Tissue Allografts. Boston, MA: Springer US (2008). p. 215–33.

[32] Gorantla VS Plock JA Davis MR Reconstructive Transplantation: Program, Patient, Protocol, Policy, and Payer Considerations, Anesthesia and Perioperative Care for Organ Transplantation. New York, NY: Springer New York (2016). p. 553–60.

[33] Schneeberger S Ninkovic M Margreiter R Hand Transplantation: The Innsbruck Experience, Transplantation of Composite Tissue Allografts. Boston, MA: Springer US (2008). p. 234–50.

[34] Weissenbacher A Hautz T Pierer G Ninkovic M Zelger BG Zelger B Hand transplantation in its fourteenth year: the innsbruck experience. Vascularized Composite Allotransplantation (2014) 1(1-2):11–21. 10.4161/23723505.2014.973798

[35] Sweeney MJ Hoffman DH Esterman MA Metabolism and biochemistry of mycophenolic acid. Cancer Res (1972) 32(9):1803–9. 4629779

[36] Bentley R Mycophenolic Acid: a one hundred year odyssey from antibiotic to immunosuppressant. Chem Rev (2000) 100(10):3801–26. 10.1021/cr990097b 11749328

[37] Williams RH Lively DH Delong DC Cline JC Sweeny MJ Mycophenolic acid: antiviral and antitumor properties. J Antibiot (1968) 21(7):463–4. 10.7164/antibiotics.21.463 4303502

[38] Suzuki S Takaku S Mori T Antitumor activity of derivatives of mycophenolic acid. J Antibiot (1976) 29(3):275–85. 10.7164/antibiotics.29.275 1262254

[39] Devyatko E Dunkler D Bohdjalian A Zuckermann A Grimm M Muehlbacher F Lymphocyte activation and correlation with IMPDH activity under therapy with mycophenolate mofetil. Clin Chim Acta (2008) 394(1-2):67–71. 10.1016/j.cca.2008.04.006 18474232

[40] Quaratino CP Messina E Spoto G Gizzi F Ruffini I Odorisio M IMP-dehydrogenase (IMPDH), hypoxanthine-guanine phosphoribosyltransferase (HGPRT) and phosphodiesterases (PDES) expression during mycophenolic acid (MPA)-induced differentiation in human neuroblastoma cell lines. Clin Biochem (1997) 30(3):275 10.1016/S0009-9120(97)87766-1 9598106

[41] Arns W Noninfectious Gastrointestinal (GI) complications of mycophenolic acid therapy: a consequence of local GI toxicity? Transplant. Proc. (2007) 39(1):88–93. 10.1016/j.transproceed.2006.10.189 17275481

[42] Gorantla VS Barker JH Jones JW Prabhune K Maldonado C Granger DK Immunosuppressive agents in transplantation: mechanisms of action and current anti-rejection strategies. Microsurgery (2000) 20(8):420–9. 10.1002/1098-2752(2000)20:8<420::AID-MICR13>3.0.CO;2-O 11150994

[43] CellCept and Myfortic: serious adverse events. Reactions Weekly (2015) 1533(1):1–1.

[44] Banga AK Transdermal and Intradermal Delivery of Therapeutic Agents. Florida, United States: CRC Press (2011).

[45] Prausnitz MR Langer R Delivery Tdrug Nat Biotechnol (2008) 26(11):1261–8. 18997767 10.1038/nbt.1504 PMC2700785

[46] Kamel R Transdermal Drug Delivery: Benefits and Challenges. J App Pharm (2016) 08(01). 10.4172/1920-4159.1000e103

[47] Münch S Wohlrab J Neubert RHH Dermal and transdermal delivery of pharmaceutically relevant macromolecules. Eur J Pharm Biopharm (2017) 119:235–42. 10.1016/j.ejpb.2017.06.019 28647443

[48] Morales JO Fathe KR Brunaugh A Ferrati S Li S Montenegro-Nicolini M Challenges and Future Prospects for the Delivery of Biologics: Oral Mucosal, Pulmonary, and Transdermal Routes. Aaps J (2017) 19(3):652–68. 10.1208/s12248-017-0054-z 28194704

[49] Benson HAE Watkinson AC Topical and Transdermal Drug Delivery. NJ, United States: John Wiley & Sons (2012).

[50] Montenegro L Puglisi G Evaluation of sunscreen safety by in vitro skin permeation studies: effects of vehicle composition. Pharmazie (2013) 68(1):34–40. 23444778

[51] Annesley TM Clayton LT Quantification of mycophenolic acid and glucuronide metabolite in human serum by HPLC-tandem mass spectrometry. Clin Chem (2005) 51(5):872–7. 10.1373/clinchem.2004.047357 15746301

[52] Lambers H Piessens S Bloem A Pronk H Finkel P Natural skin surface pH is on average below 5, which is beneficial for its resident flora. Int J Cosmet Sci (2006) 28(5):359–70. 10.1111/j.1467-2494.2006.00344.x 18489300

[53] Shah JC Analysis of permeation data: evaluation of the lag time method. Int J Pharm (1993) 90(2):161–9. 10.1016/0378-5173(93)90152-6

[54] Thompson RH Whittaker VP The esterases of skin. Biochem J (1944) 38(4):295–9. 10.1042/bj0380295 16747798 PMC1258089

[55] Handjani F Aghaei S Moezzi I Saki N Topical mycophenolate mofetil in the treatment of vitiligo: a pilot study. Dermatol Pract Concept (2017) 7(2):31–3. 10.5826/dpc.0702a06 PMC5424659 28515990

[56] Mason J Mason AR Cork MJ Topical preparations for the treatment of psoriasis: a systematic review. Br J Dermatol (2000) 142(3):351–64. 10.1046/j.0007-0963.2002.04713.x 11952534

[57] Jung KE Lee YJ Ryu YH Kim JE Kim HS Kim BJ Effects of topically applied rapamycin and mycophenolic acid on TNCB-induced atopic dermatitis-like skin lesions in NC/Nga mice. Int Immunopharmacol (2015) 26(2):432–8. 10.1016/j.intimp.2015.03.007 25794646

[58] Shoji Y Fukumura T Kudo M Yanagawa A Shimada J Mizushima Y Effect of topical preparation of mycophenolic acid on experimental allergic contact dermatitis of guinea-pigs induced by dinitrofluorobenzene. J Pharm Pharmacol (1994) 46(8):643–6. 10.1111/j.2042-7158.1994.tb03874.x 7815276

[59] Geilen CC Mrowietz U Lack of efficacy of topical mycophenolic acid in psoriasis vulgaris. J Am Acad Dermatol (2000) 42(5 Pt 1):837–40. 10.1067/mjd.2000.105561 10775867

[60] Finnin BC Morgan TM Transdermal penetration enhancers: applications, limitations, and potential. J Pharm Sci (1999) 88(10):955–8. 10.1021/js990154g 10514338

[61] Amnuaikit T Songkram C Pinsuwan S Enhancement of mycophenolate mofetil permeation for topical use by eucalyptol and N-Methyl-2-pyrrolidone. Scientifica (2016) 2016(3):1–10. 10.1155/2016/9672718 PMC4812526 27069715

[62] Haigh JM Smith EW The selection and use of natural and synthetic membranes for in vitro diffusion experiments. European Journal of Pharmaceutical Sciences (1994) 2(5-6):311–30. 10.1016/0928-0987(94)90032-9

[63] Barbero AM Frasch HF Pig and guinea pig skin as surrogates for human in vitro penetration studies: a quantitative review. Toxicol In Vitro (2009) 23(1):1–13. 10.1016/j.tiv.2008.10.008 19013230

[64] Frasch HF Barbero AM A paired comparison between human skin and hairless guinea pig skin in vitro permeability and lag time measurements for 6 industrial chemicals. Cutan Ocul Toxicol (2009) 28(3):107–13. 10.1080/15569520902950474 19552540

[65] Todo H Transdermal permeation of drugs in various animal species. Pharmaceutics (20172017) 9(4):33 10.3390/pharmaceutics9030033 PMC5620574 28878145

[66] Praça FSG Medina WSG Eloy JO Petrilli R Campos PM Ascenso A Evaluation of critical parameters for in vitro skin permeation and penetration studies using animal skin models. Eur J Pharm Sci (2018) 111:121–32. 10.1016/j.ejps.2017.09.034 28951120

[67] Ahlstrom LA Cross SE Mills PC The effects of freezing skin on transdermal drug penetration kinetics. J Vet Pharmacol Ther (2007) 30(5):456–63. 10.1111/j.1365-2885.2007.00879.x 17803739

[68] Barbero AM Frasch HF Effect of frozen human epidermis storage duration and cryoprotectant on barrier function using two model compounds. Skin Pharmacol Physiol (2016) 29(1):31–40. 10.1159/000441038 26606593 PMC4742402

[69] Ge L Huang Z Wei H Skin Graft Preservation. Skin Grafts - In*dications, Applications and Current Research*. InTech (2011).

[70] Zschocke I Mrowietz U Karakasili E Reich K Non-adherence and measures to improve adherence in the topical treatment of psoriasis. J Eur Acad Dermatol Venereol (2014) 28(Suppl 2):4–9. 10.1111/jdv.12445 24684738

[71] Eastman WJ Malahias S Delconte J Dibenedetti D Assessing attributes of topical vehicles for the treatment of acne, atopic dermatitis, and plaque psoriasis. Cutis (2014) 94(1):46–53. 25101344

[72] Cai S Yang Q Bagby TR Forrest ML Lymphatic drug delivery using engineered liposomes and solid lipid nanoparticles. Adv Drug Deliv Rev (2011) 63(10-11):901–8. 10.1016/j.addr.2011.05.017 21712055 PMC3164476

[73] Ali Khan A Mudassir J Mohtar N Darwis Y Advanced drug delivery to the lymphatic system: lipid-based nanoformulations. Int J Nanomedicine (2013) 8(1):2733–44. 10.2147/IJN.S41521 23926431 PMC3732201

